# Structural Adaptations of Bacterial Grx3 to Temperature: Pro29 Is Essential for Cold Adaptation in *Sphingomonas* sp. Grx3

**DOI:** 10.1021/acsomega.5c03414

**Published:** 2025-05-26

**Authors:** Luyen Vu, ChangWoo Lee

**Affiliations:** Department of Biomedical Science and Center for Bio-Nanomaterials, 37975 Daegu University, Gyeongsan 38453, South Korea

## Abstract

Bacterial glutaredoxin 3 (Grx3) is a class I oxidoreductase with a canonical thioredoxin (Trx) fold, yet its thermal adaptation mechanisms remain unclear. We investigated cold adaptation in the psychrophilic ortholog from the Arctic bacterium *Sphingomonas* sp. (SpGrx3). While mesophilic orthologs, such as Escherichia coli Grx3 (EcGrx3), typically feature an α1−β2 salt bridge (Lys19-Glu31), SpGrx3 lacks this bridge due to Leu19, although Glu31 is conserved. The L19K mutation in SpGrx3 failed to form the salt bridge, yielding the least stable mutant. However, the introduction of a P29F or P29Y substitution in the α1 loop in combination with L19K restored the salt bridge and significantly enhanced both the melting temperature and stability. Phe29 stabilizes the structure via hydrophobic interactions, enhancing substrate affinity, while Tyr29 enhances catalytic rates through hydrogen bonding. Conversely, the reciprocal F29P substitution in EcGrx3 disrupted the salt bridge and markedly reduced its melting temperature. Notably, K19L/F29P, which mimics the SpGrx3 wild-type (WT) configuration, increased melting temperature via Leu19 hydrophobic interactions, similar to F29Y with the salt bridge. These results underscore the crucial role of Phe or Tyr at position 29 in forming the Lys19-Glu31 salt bridge in warmer-temperature orthologs and suggest that the transition to Pro is a critical adaptation in psychrophilic orthologs. This study provides new insights into Grx3′s structural adaptations to varying thermal habitats through diverse α1−β2 interactions.

## Graphical abstract

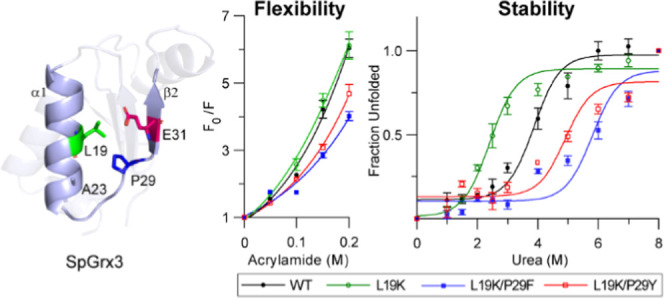

## 1 Introduction

Glutaredoxins (Grxs) are small, ubiquitous redox proteins with a Trx fold that are evolutionarily conserved.^1–5^ They protect cells from oxidative stress by catalyzing glutathione (GSH)-dependent redox reactions—both through glutathionylation and disulfide reduction in concert with glutathione reductase and NADPH.^1,6,7^ There are six classes of Grxs, with class I and II being common.^8^ Class I Grxs, characterized by a CxxC/S active-site motif, function as GSH-dependent oxidoreductases, while class II Grxs, with a CGFS active-site motif, act as Fe–S cluster transferases and possess a five amino-acid extension before the N-terminal active-site Cys.^8^

Among the class I Grxs, bacterial Grx3 (approximately 85–95 amino acids) serves a key hydrogen donor for ribonucleotide reductase and is essential for maintaining cellular redox balance.^1,9–11^ Grx3 adopts the canonical Trx fold—a four-stranded β-sheet flanked by three α-helices—in which the central β-sheet divides the hydrophobic core into aromatic and aliphatic clusters.^12–14^ Although Grx3 shares the Trx fold, it exhibits greater conformational flexibility than Trx,^15^ partly because it lacks one α-helix and one β-strand found in Trx, which functions as a substrate for Trx reductase.^13,14,16^ In contrast, the aromatic cluster in Trx features a tetrahedral arrangement of aromatic residues, with two positioned in the β2 and β4 strands and two in the α1 and short α3 helices.^13,14,16^

In Grx3, aromatic residues in the corresponding β1 and β3 strands are maintained, but two key alterations occur—a Phe-to-Tyr substitution in the β1 strand and a Phe-to-Arg replacement in the short α2 helix—reducing hydrophobicity in the central β-sheet and α2 region.^15,17–19^ Although the GSH binding site remains conserved across temperatures,^18,20,21^ some psychrophilic orthologs exhibit reduced GSH binding due to an Arg-to-Tyr substitution in the α2 helix that shifts binding from a salt bridge to hydrogen bonding.^20,22^

Given that these structural changes occur on the aromatic cluster side (namely, Tyr7 and Phe58 [SpGrx3 numbering]),^15,20^ we investigated temperature adaptation mechanisms on the aliphatic cluster side (Figure 1). In mesophilic (e.g., EcGrx3) and thermophilic orthologs, an α1−β2 salt bridge is typically formed by a Lys19-Glu31 pair, with Glu31 conserved (Figure 1A,D), whereas psychrophilic orthologs (e.g., SpGrx3) lack this bridge (Figure 1A–C). In some thermophilic orthologs, such as TtGrx3 (Thermochromatium tepidum), Lys is often replaced by Arg (Figures 1A and S1), while SpGrx3 features Leu, thereby disrupting the salt bridge. We first introduced an L19K substitution in SpGrx3 to restore the salt bridge; when that failed, we explored additional structural adaptations.

**Figure 1:**
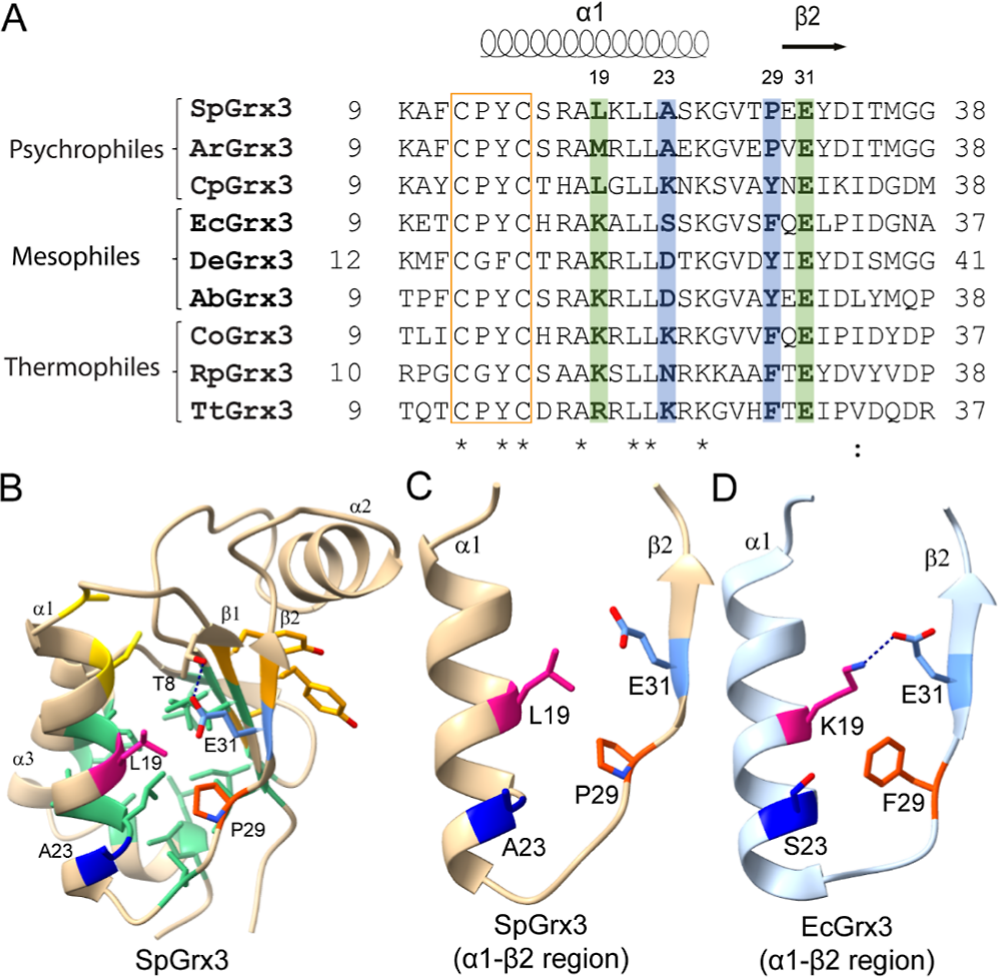
Sequence and structure comparison of Grx3 orthologs. (A) Multiple sequence alignment of Grx3 members from various temperature environments. The active-site CXXC motif is highlighted in an orange box. Residues at positions 19 and 31, forming the α1−β2 salt bridge in mesophilic and thermophilic species, are marked in green. Residues at positions 23 and 29, forming the intraloop hydrogen bond in some mesophilic and psychrophilic orthologs, are marked in blue. Psychrophilic Grx3s: SpGrx3 (*Sphingomonas* sp. PAMC 26621, NCBI ID: WP_010217562.1), ArGrx3 (*Arthrobacter* sp. TPD3018, NCBI ID: PVE52959.1), CpGrx3 (Colwellia piezophile, NCBI ID: WP_019027132.1); Mesophilic Grx3s: EcGrx3 (Escherichia coli K-12, PDB ID: 1FOV), DeGrx3 (*Desulfuromonadales* bacterium, NCBI ID: NIQ93024.1), AbGrx3 (Azospirillum brasilense, NCBI ID: WP_200477686.1); Thermophilic Grx3s: CoGrx3 (Chromatium okenii, NCBI ID: WP_105074337.1), RpGrx3 (Rhodopseudomonas palustris, NCBI ID: WP_110787285.1), TtGrx3 (Thermochromatium tepidum ATCC 43061, NCBI ID: QGU33019.1). (B) Structural model of SpGrx3. The two catalytic Cys residues are shown in yellow. Residues in the aliphatic cluster are depicted in green, and those in the aromatic cluster in orange. (C,D) Enlarged views of the α1−β2 regions of SpGrx3 and EcGrx3, respectively.

This led us to focus on Pro29 in the α1 loop of SpGrx3, despite the fact that Pro’s rigidity is typically minimized in cold-adapted enzymes.^23^ In warmer-temperature orthologs, an aromatic residue (Phe or Tyr) occupies this position, though it is not part of the conserved aromatic cluster. We introduced P29F and P29Y substitutions—with and without the L19K mutation—and also mutated Ala23 to Asp or Lys (A23D, A23K) to assess the role of Tyr29 in intraloop hydrogen bonding. Reciprocal mutations were performed on EcGrx3 to disrupt the Lys19-Glu31 salt bridge (K19A, K19L) and to substitute the residue at position 29 (F29P, F29Y). We also analyzed the WT configuration of SpGrx3 (K19L/F29P).

Cold-adapted enzymes generally exhibit greater flexibility due to features such as fewer intramolecular bonds, loop extensions, higher Gly/Pro and Lys/Arg ratios, and enhanced active-site accessibility.^23–27^ Overall, our findings underscore that diverse α1−β2 structural adaptations are crucial for Grx3′s temperature adaptation.

## 2 Materials and Methods

### 2.1 Materials

The strain *Sphingomonas* sp. PAMC 26621 was provided by the Polar and Alpine Microbial Collection at the Korea Polar Research Institute (Incheon, South Korea).^28^ The pET28 expression vector was obtained from Novagen (Madison, WI, USA), while HisTrap, Capto Q, and Superdex 200 prep grade XK16 columns were purchased from Cytiva (Marlborough, MA, USA). The *n*Pfu-Forte polymerase and DpnI were obtained from Enzynomics (Daejeon, South Korea), and NADPH from Tokyo Chemical Industry (Tokyo, Japan). Unless otherwise noted, all other reagents were purchased from Sigma-Aldrich (St. Louis, MO, USA).

### 2.2 Site-Directed Mutagenesis

The *spgrx3* gene (NCBI ID: NZ_AIDW01000021.1:c56439-56182) was amplified by polymerase chain reaction (PCR) from the genome of *Sphingomonas* sp. PAMC 26621. The *ecgrx3* gene (NCBI ID: 948132) was synthesized by Bioneer (Daejeon, South Korea). After inserting the *spgrx3* and *ecgrx3* genes into the pET28 vector, the resulting pET28-*spgrx3* and pET28-*ecgrx3* constructs were used as templates for PCR-based site-directed mutagenesis using *n*Pfu-Forte polymerase (PCR primers listed in Table S1). The PCR products were then incubated with DpnI at 37 °C for 1 h to digest the template plasmids prior to transformation into Escherichia coli BL21 (DE3) for protein expression. Plasmid sequences were validated by Sanger sequencing.

### 2.3 Bioinformatics

The three-dimensional structure models were generated using AlphaFold2,^29^ incorporating data from the AlphaFold Protein Structural Database. ChimeraX was used for protein structure visualization and amino acid interaction analysis.^30^ Multiple sequence alignments were performed with Clustal Omega.^31^

### 2.4 Protein Expression and Purification

A single colony of E. coli BL21­(DE3) containing recombinant WT and mutant SpGrx3 and EcGrx3 were grown overnight in Luria–Bertani (LB) medium with 100 μg/mL kanamycin. The overnight culture was then inoculated into a flask with 200 mL of LB medium and incubated at 37 °C, 225 rpm. When the culture reached an optical density of 0.6–0.8, isopropyl β-d-1-thiogalactopyranoside was added to a final concentration of 1 mM. Cultures were then incubated for an additional 24 h at 25 °C for SpGrx3 and 5 h at 30 °C for EcGrx3. Cells were harvested by centrifugation, washed, resuspended in buffer A (50 mM Tris·HCl, 300 mM NaCl, 5 mM imidazole, 0.1 mM EDTA, pH 8.0), and disrupted by sonication on ice. After centrifugation at 10,000 *g* for 10 min at 4 °C, the supernatant was loaded onto a 5 mL HisTrap column pre-equilibrated with buffer A using an AKTA go system (Cytiva). Nonspecific proteins were removed with buffer A supplemented with 45 mM imidazole, and the target protein was eluted with a linear gradient of 45–300 mM imidazole in buffer B (50 mM Tris·HCl, 50 mM NaCl, 300 mM imidazole, 0.1 mM EDTA, pH 8.0). Fractions containing the recombinant proteins were pooled and applied to a 5 mL Capto Q column, equilibrated with buffer C (50 mM Tris·HCl, 50 mM NaCl, pH 8.0). Target proteins were pooled, exchanged into buffer D (100 mM Tris·HCl, pH 8.0) with 5% glycerol, and stored at –80 °C for further use.

The molecular weights (MW) of SpGrx3 proteins were determined by size-exclusion chromatography using a Superdex 200 column in buffer C, calibrated with a protein standard mix comprising cytochrome c (12.4 kDa), carbonic anhydrase (29 kDa), albumin (66 kDa), alcohol dehydrogenase (150 kDa), and β-amylase (200 kDa).

### 2.5 Protein Thermal Shift Analysis

A thermal shift assay was performed using SYPRO Orange on an Applied Biosystems StepOnePlus real-time PCR instrument (Thermo Fisher Scientific, Waltham, MA, USA) with a continuous heating ramp from 25 to 99 °C at 1% per sec. SpGrx3 and EcGrx3 were added at a final concentration of 4 mg/mL to buffer D, with 25× and 250× SYPRO Orange, respectively, in a total volume of 20 μL. The melting temperature (*T*_m_), indicating the temperature at which 50% of the protein is denatured, was determined using Protein Thermal Shift software v1.4 (Applied Biosystems).

### 2.6 Fluorescence Spectroscopy

Intrinsic protein fluorescence was measured to evaluate urea-induced unfolding and acrylamide quenching using a Scinco FS-2 fluorescence spectrometer (Seoul, South Korea). Since SpGrx3 lacks Trp residues, the excitation wavelength was set at 275 nm and emission was recorded from 285 to 350 nm. The conformational flexibility of SpGrx3 and its mutants were assessed by fluorescence quenching with acrylamide (0–0.2 M). A 250 μg sample of protein was incubated with 120 μL of buffer D containing acrylamide for 2 min at 25 °C, then fluorescence was measured. Stern–Volmer plots were generated from the ratio of fluorescence without acrylamide (*F*_0_) to that with acrylamide (*F*), applying the equation^32^

$$F_{0}/F=1+K_{sv}\left[Q\right]$$

where [*Q*] is the acrylamide concentration and *K*_sv_ is the Stern–Volmer constant determined from the slope of the plot. Data were plotted using GraphPad Prism (San Diego, CA, USA).

Urea-induced unfolding of SpGrx3 was performed on 150 μg of protein incubated with urea (0–8 M) in buffer E (100 mM potassium phosphate, 100 mM NaCl, pH 7.4) at 25 °C for 30 min. The equilibrium constant (*K*_eq_) was used to calculate the Gibbs free energy Δ*G*_H_2_O_^°^ = −*RT* ln (*K*_eq_), where *R* is the gas constant (J·K^–1^ mol^–1^) and *T* is the temperature (K). A linear relationship between Δ*G* and urea concentration was observed, and fractional unfolding was plotted using GraphPad Prism.

### 2.7 Kinetic Analysis

Grx3 activity was measured using hydroxyethyl disulfide (HED) assay.^33^ The 1 mL assay mixture contained 1 mM GSH, 0.4 mM NADPH, 0.3 μg yeast glutathione reductase, 2 mM EDTA, 0.1 mg BSA, 2.5 mM HED, and 20 μg SpGrx3 protein in buffer D. The mixture was incubated at various temperatures for 5 min, and absorbance at 340 nm was recorded using a Shimadzu UV-1800 spectrophotometer (Kyoto, Japan) to determine the optimal temperature. Enzyme kinetics for SpGrx3 and its mutants were evaluated at GSH concentrations ranging from 0.3 to 1 mM at their respective optimal temperatures (Figure S2). The Michaelis constant (*K*_m_) and catalytic rate (*k*_cat_) values were estimated using Lineweaver–Burk plots generated in GraphPad Prism.

### 2.8 Far-UV Circular Dichroism (CD) Spectroscopy

Protein samples at 1 mg/mL in buffer D were analyzed using a JASCO J-1500 spectropolarimeter (Tokyo, Japan) at the Korea Basic Science Institute (Ochang, South Korea). CD spectra were plotted as residual ellipticity (mdeg) versus wavelength (nm), and the α-helix and β-strand contents were calculated using the K2D3 server.

### 2.9 Molecular Dynamics (MD) Simulation

MD simulations of WT and mutant SpGrx3 and EcGrx3 were performed using GROMACS at BioCode Ltd. (Liverpool, Merseyside, UK). Initially, proteins were prepared by removing water molecules and ligands, then solvated in box with water replaced by monatomic ions. Energy minimization was performed to eliminate steric clashes and optimize molecular geometry. The MD simulation was conducted for 200 ns under constant temperature (300 K) and constant pressure (*NPT* ensemble). Postsimulation, the root-mean-square deviation (RMSD) and root-mean-square fluctuation (RMSF) of C_α_ atoms were calculated.

## 3 Results

### 3.1 Protein Expression and Purification

Recombinant SpGrx3 WT and mutants, each with an N-terminal 6 × His-tag, were expressed as soluble proteins in E. coli BL21­(DE3) and purified to apparent homogeneity by nickel-chelate affinity chromatography followed by Capto Q anion-exchange chromatography, yielding a single SDS-PAGE band (Figure S3). Size-exclusion chromatography confirmed that the proteins are monomers, with an MW of 11.41 kDa (Figure S4).

### 3.2 *T*_m_ Analysis

We measured the *T*_m_ values of SpGrx3 WT and mutants using a SYPRO Orange thermal shift assay (Table 1, Figure S5). The WT protein had a *T*_m_ of 52.8 °C. The L19K mutant—designed to restore the α1−β2 (Lys19-Glu31) salt bridge—showed a slightly lower *T*_m_ of 51 °C, suggesting salt bridge failure and a destabilizing effect from the loss of hydrophobic Leu. Sequence comparison indicates that psychrophilic SpGrx3 and its orthologs contain Pro at position 29 in the α1 loop, whereas warmer orthologs (e.g., EcGrx3) feature Phe or Tyr (Figure 1A). Replacing Pro29 with Phe or Tyr (P29F and P29Y) raised the *T*_m_ by approximately 6.3 °C (to 57.3 and 57.4 °C, respectively). Moreover, the double mutants L19K/P29F and L19K/P29Y further increased the *T*_m_ by 14.6 and 7.8 °C above WT, reaching 67.4 and 60.6 °C, respectively.

**Table 1:** *T*_m_ Values of SpGrx3 WT, EcGrx3 C66Y, and Their Mutants

SpGrx3	*T*_m_ (°C)	Δ*T*_m_ (°C)^a^	EcGrx3	*T*_m_ (°C)	Δ*T*_m_ (°C)^a^
WT	52.8 ± 0.1	0	C66Y	66.6 ± 0.5	0
L19K	51.0 ± 0.2	–1.8	K19A	55.6 ± 0.3	–11.0
P29F	57.3 ± 0.1	4.5	K19L	60.1 ± 0.1	–6.5
P29Y	57.4 ± 0.1	4.6	F29P	52.6 ± 0.1	–14.0
L19K/P29F	67.4 ± 0.1	14.6	F29Y	57.7 ± 0.1	–8.9
L19K/P29Y	60.6 ± 0.1	7.8	K19L/F29P	57.8 ± 0.5	–8.8
A23D/P29Y	60.9 ± 0.4	8.1			
A23K/P29Y	65.1 ± 0.1	12.3			
L19K/A23D/P29Y	61.7 ± 0.1	8.9			
L19K/A23K/P29Y	66.5 ± 0.1	13.7			
L19K/A23K/P29F	64.4 ± 0.1	11.6			

a Changes in *T*_m_ value for mutants compared to the WT: Δ*T*_m_ = *T*_m_ (mutant) – *T*_m_ (WT or C66Y). SpGrx3 WT and EcGrx3 C66Y were used as baselines in this study. Data presented are the mean ± SD of three experiments.

Given the 6.8 °C difference between L19K/P29F and L19K/P29Y, we further examined the role of Tyr29, which may form an intraloop hydrogen bond with Asp or Lys at position 23 in some psychrophilic and mesophilic orthologs, but not in thermophilic ones (e.g., CpGrx3, DeGrx3, AbGrx3; Figure 1A). Triple mutants L19K/A23D/P29Y and L19K/A23K/P29Y exhibited *T*_m_ values of 61.7 and 66.5 °C, respectively, while the corresponding double mutants with only the intraloop hydrogen bond (A23D/P29Y and A23K/P29Y) showed slightly lower *T*_m_ values (60.9 and 65.1 °C). Notably, L19K/A23K/P29F, with an unpaired Lys23, had a *T*_m_ of 64.4 °C—3 °C lower than L19K/P29F mutant—suggesting that an unpaired Lys23 destabilizes the hydrophobic aliphatic cluster.

These results indicate that the P29F and P29Y substitutions in the α1 loop are critical for forming the Lys19-Glu31 salt bridge. Phe29 enhances thermal stability via hydrophobic interactions, while Tyr29 stabilizes the structure through hydrogen bonding. Moreover, the small (1 °C) *T*_m_ difference between the salt bridge and Leu19 hydrophobic interactions—both paired with Tyr29 hydrogen bond—suggests that each contributes similarly to stabilizing the aliphatic cluster.

### 3.3 Conformational Stability Assessment

To assess the conformational stability of SpGrx3 WT and mutants, we performed urea-induced protein unfolding experiments at 25 °C using fluorescence spectroscopy (Table S2, Figure 2). L19K showed the lowest urea midpoint at 2.4 M, compared to 3.1 M for SpGrx3 WT. In contrast, other mutants containing Phe29 or Tyr29 showed higher stability than WT, with urea midpoints ranging from 4.6 to 6.1 M. Consistent with the *T*_m_ value results, Phe29 provides a stronger stabilizing effect than Tyr29 under urea treatment in both single and double mutants: P29F (5.0 M) > P29Y (4.6 M) and L19K/P29F (5.8 M) > L19K/P29Y (5.1 M). Notably, mutants with the Asp23-Tyr29 hydrogen bond (A23D/P29Y, L19K/A23D/P29Y) show enhanced stability compared to those with the Lys23-Tyr29 pair (A23K/P29Y, L19K/A23K/P29Y). This is evidenced by the triple mutant L19K/A23D/P29Y, which, with both the salt bridge and the intraloop hydrogen bond, reached the highest urea midpoint to 6.1 M. Overall, these findings indicate that disrupting the hydrophobic interactions in the aliphatic cluster, particularly involving Leu19, reduces SpGrx3′conformational stability. The results also show that the α1 loop aromatic residue—whether Phe29 through hydrophobic interactions or Tyr29 through hydrogen bonding—can enhance SpGrx3′s structural integrity.

**Figure 2:**
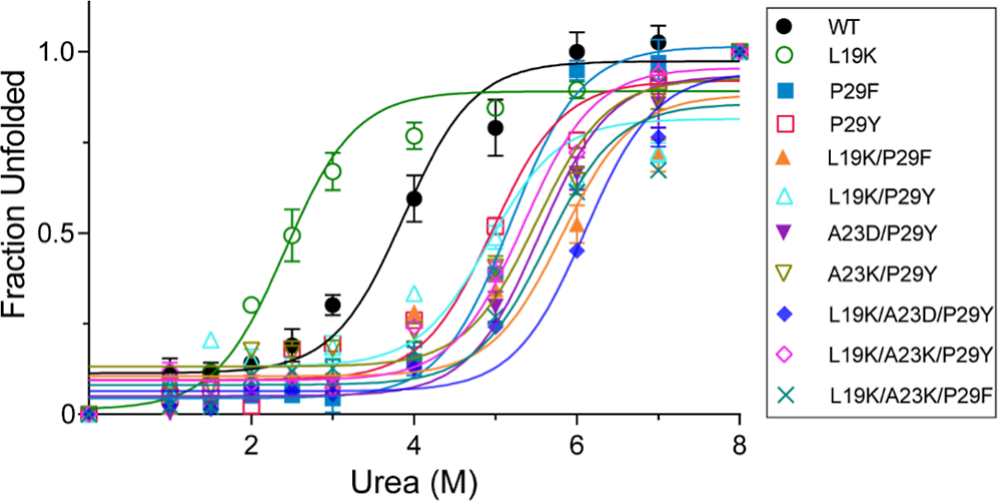
Conformational stability of SpGrx3 WT and mutants. Fluorescence spectra were recorded after 30 min incubation at 25 °C with 0–8 M urea (excitation at 275 nm). Data represents the mean ± SD of three experiments.

### 3.4 Conformational Flexibility Evaluation

We assessed the conformational flexibility of SpGrx3 WT and mutants using acrylamide quenching of intrinsic protein fluorescence (excitation at 275 nm, as SpGrx3 lacks Trp residues). The L19K mutant exhibited slightly higher flexibility than WT, whereas mutants with the P29F or P29Y substitution showed increased rigidity (Table S3, Figure 3). Notably, L19K/P29F was the most rigid, consistent with its highest *T*_m_ value. However, mutants forming an Asp23-Tyr29 hydrogen bond (A23D/P29Y, L19K/A23D/P29Y) were more flexible than those with a Lys23-Tyr29 hydrogen bond (A23K/P29Y, L19K/A23K/P29Y), despite improved urea stability. Overall, these results underscore the importance of Leu19 hydrophobic interactions and the aromatic residue in the α1 loop for SpGrx3 stability—Phe29 enhances stability through hydrophobic interactions, while Tyr29 primarily stabilizes via hydrogen bonding.

**Figure 3:**
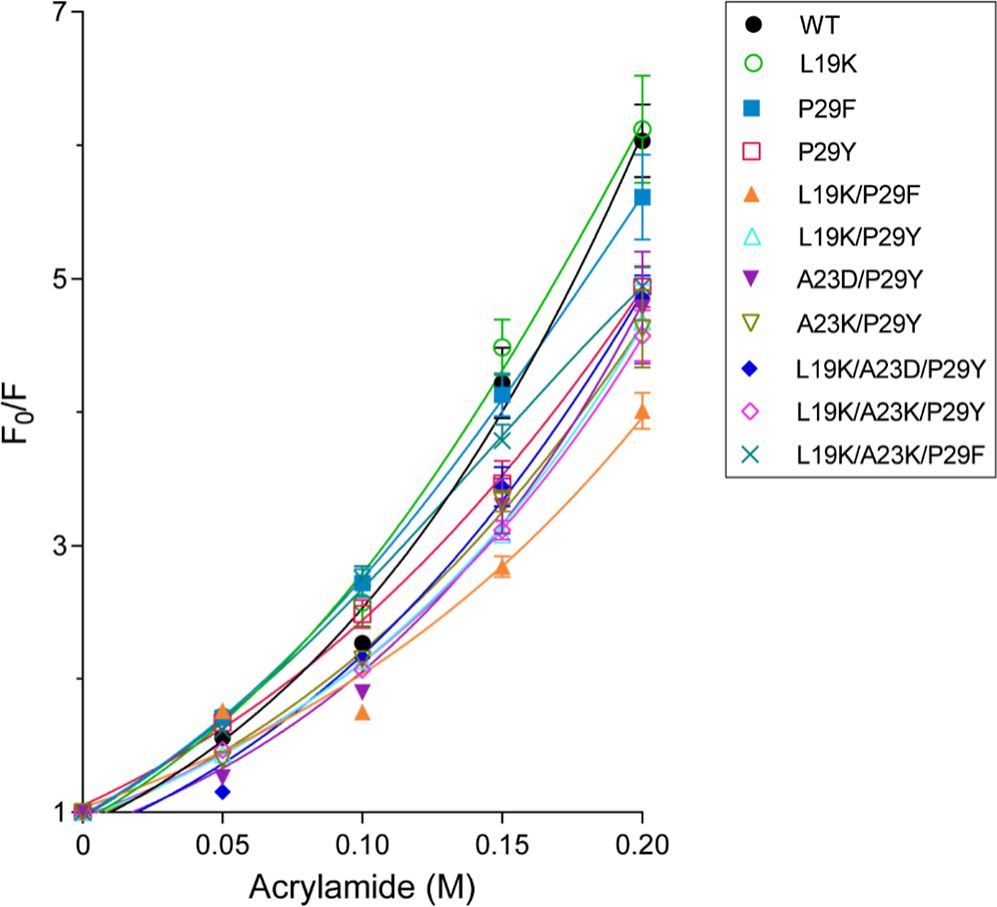
Acrylamide Stern–Volmer plot of SpGrx3 WT and mutants. Fluorescence was recorded as the maximum intensity in the absence (*F*_0_) and presence (*F*) of acrylamide, and the ratio *F*_0_/*F* was plotted. Data represents the mean ± SD of three experiments.

### 3.5 Secondary Structure Analysis

We analyzed the secondary structure of SpGrx3 WT and its mutants by far-UV CD spectroscopy (Figure 4). The WT showed 35% α-helix and 11% β-strand content (Table S4). In contrast, all mutants showed a marked reduction in α-helix content, ranging from 5 to 25%. Mutants with the P29F substitution (P29F, L19K/P29F, L19K/A23K/P29F) had particularly low α-helix content (11%, 6%, and 10%, respectively). Although the single substitutions P29F and P29Y resulted in similar α-helix content (11%), the L19K/P29F mutant exhibited the lowest value overall. These findings suggest that changes in the α1−β2 region alter SpGrx3′s secondary structure, with the salt bridge formation and Phe29 hydrophobic interactions contributing to reduced α-helix content.

**Figure 4:**
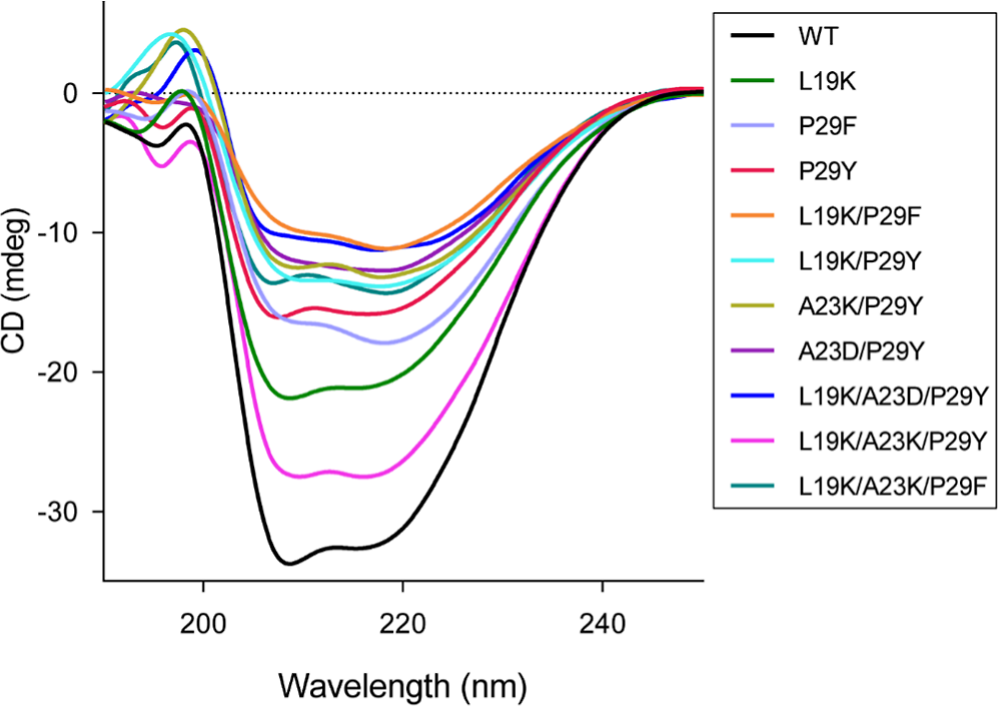
Far-UV CD spectra of SpGrx3 WT and mutants at 25 °C.

### 3.6 Enzyme Kinetics

Enzyme kinetics experiments were performed at the optimal temperatures using the HED assay with GSH as the substrate and NADPH as the cofactor (Table 2). SpGrx3 WT had a *K*_m_ of 4.3 mM and a *k*_cat_ of 155.3 min^–1^. The L19K mutant, which destabilizes the protein, showed slightly higher values (4.6 mM and 164.2 min^–1^, respectively) compared to WT. Mutants with Phe29 or Tyr29 had lower *K*_m_ values (2.6 and 2.3 mM, respectively) and the lowest *k*_cat_ values (66.3, 64.5 min^–1^, respectively), likely due to Phe29’s hydrophobic interactions. Meanwhile, P29Y and L19K/P29Y exhibited kinetic parameters similar to WT, with *K*_m_ values of 4.3 and 4.0 mM, and *k*_cat_ values of 150.6 and 133.2 min^–1^, respectively.

**Table 2:** Kinetic Parameters of SpGrx3 WT and Mutants^a^

SpGrx3	*K*_m_ (mM)	*k*_cat_ (min^–1^)	*k*_cat_/*K*_m_ (min^–1^/mM)
WT	4.3 ± 0.8	155.3 ± 36.7	36.1 ± 2.3
L19K	4.6 ± 0.8	164.2 ± 38.9	35.6 ± 4.1
P29F	2.6 ± 0.2	66.3 ± 8.4	25.1 ± 1.2
P29Y	4.3 ± 0.6	150.6 ± 26.3	35.0 ± 1.6
L19K/P29F	2.3 ± 0.7	64.5 ± 24.9	27.9 ± 3.3
L19K/P29Y	4.0 ± 0.3	133.2 ± 15.3	33.0 ± 1.0
A23D/P29Y	5.7 ± 0.3	419.0 ± 50.9	72.9 ± 4.9
A23K/P29Y	4.0 ± 0.5	268.7 ± 43.9	67.2 ± 8.3
L19K/A23D/P29Y	3.5 ± 0.4	118.0 ± 20.2	33.4 ± 1.7
L19K/A23K/P29Y	2.4 ± 0.7	67.5 ± 23.3	27.8 ± 1.1
L19K/A23K/P29F	4.5 ± 1.2	323.4 ± 70.3	73.3 ± 6.4

a Data presented are the means ± SD of three experiments.

Mutants with only the intraloop hydrogen bond (A23D/P29Y and A23K/P29Y) showed 2.7- and 1.7-fold higher *k*_cat_ values (419.0 and 268.7 min^–1^) with *K*_m_ values close to WT (5.7 and 4.0 mM). Adding the salt bridge to these mutants (L19K/A23D/P29Y, L19K/A23K/P29Y) further reduced *K*_m_ values to 3.5 and 2.4 mM, while decreasing *k*_cat_ values to 118.0 and 67.5 min^–1^. Notably, L19K/A23K/P29F, with an unpaired Lys23, exhibited a significant *k*_cat_ increase (323.4 min^–1^) and a WT-like *K*_m_ (4.5 mM). These results suggest that Phe or Tyr at position 29 plays a key role in modulating substrate affinity: Phe29 enhances stability through hydrophobic interactions, while Tyr29 primarily stabilizes via hydrogen bonding. Notably, L19K alone had little impact on enzyme kinetics despite its significant stabilizing effect.

### 3.7 MD Simulations of SpGrx3 WT and Mutants

MD Simulations of SpGrx3 WT and four mutants (L19K, L19K/P29F, L19K/A23D/P29Y, L19K/A23K/P29Y) examined how Phe29 hydrophobic interactions and the Tyr29 intraloop hydrogen bond affect conformational flexibility (Figure 5). RMSD analysis of C_α_ atoms over the first 100 ns showed significant fluctuations, with WT being the most flexible. By 200 ns, WT and mutants stabilized, reaching similar RMSD values (1.2–1.7 Å). RMSF analysis showed that the loops, α1 helix, and α3 helix (forming the aliphatic cluster) experienced the greatest fluctuation. Gly37 exhibited the highest RMSF values: WT (2.3 Å) and L19K (2.4 Å) had the largest fluctuations, whereas L19K/P29F (2.0 Å), L19K/A23D/P29Y (2.1 Å), and L19K/A23K/P29Y (1.9 Å) were slightly less flexible. These results indicate that the α1 loop’s hydrophobic and hydrogen-bond interactions, along with the Lys19-Glu31 salt bridge, play a key role in modulating SpGrx3′s flexibility, particularly in the aliphatic cluster.

**Figure 5:**
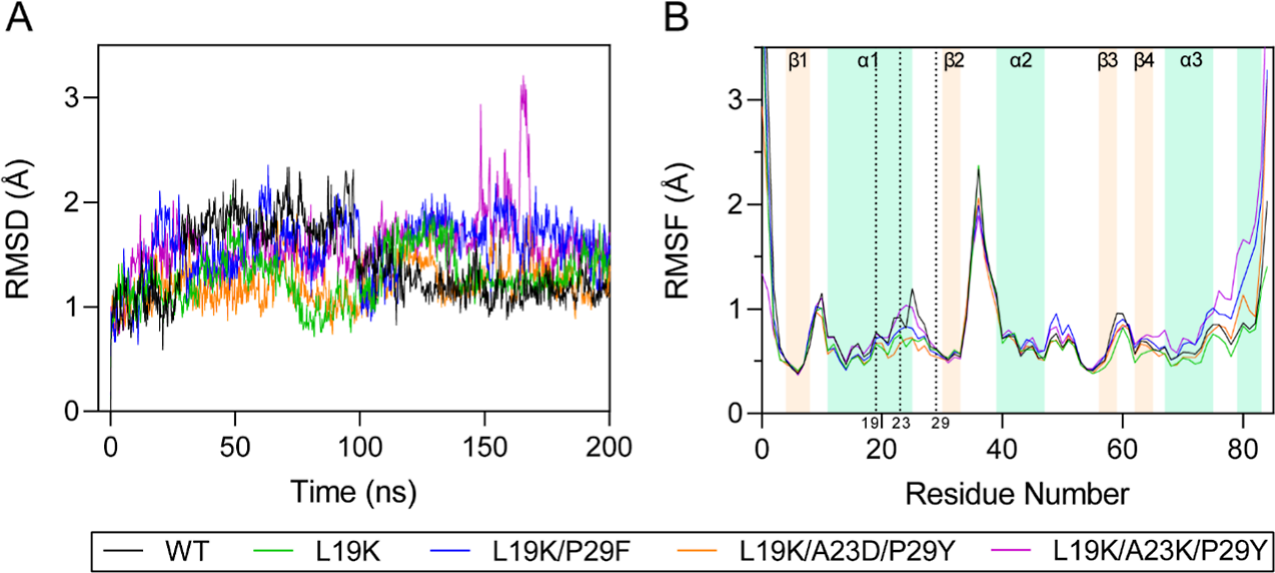
RMSD and RMSF analysis of SpGrx3 WT and mutants. (A) *C*_α_ RMSD over time. (B) *C*_α_ RMSF per residue, with secondary structure elements annotated.

### 3.8 *T*_m_ Analysis of EcGrx3 Reciprocal Mutants

To assess the role of the Lys19-Glu31 salt bridge and Phe29 in EcGrx3 stability, we introduced reciprocal mutations using the EcGrx3 C66Y mutant as a template. The C66Y substitution was used to prevent disulfide-bonded oligomers or GSH mixed disulfide bonds upon GSSG oxidation.^6,34,35^ EcGrx3 C66Y and its mutants were purified to apparent homogeneity (Figure S6), and their *T*_m_ values were measured using SYPRO Orange thermal shift assay (Table 1, Figure S7). C66Y had a *T*_m_ of 66.6 °C (we omitted the C66Y designation in mutant forms for simplicity). K19A and K19L showed *T*_m_ values of 55.6 and 60.1 °C, respectively, while F29P and K19L/F29P had *T*_m_ values of 52.6 and 57.8 °C. F29Y exhibited a *T*_m_ of 57.7 °C—8.9 °C lower than C66Y. These data indicate that each mutation (K19A, K19L, F29P, K19L/F29P) disrupts the salt bridge. The higher *T*_m_ values of K19L compared to K19A, and of K19L/F29P relative to F29P, confirms that Leu19 hydrophobic interactions contribute to thermal stability, as seen in SpGrx3. In contrast, F29Y retains the salt bridge but exhibits lower stability than C66Y, likely because Tyr provides weaker hydrophobic interactions than Phe.

### 3.9 Secondary Structure Analysis for EcGrx3 Reciprocal Mutants

The secondary structure of EcGrx3 C66Y and its mutants were analyzed using far-UV CD spectroscopy (Table S4, Figure 6). C66Y exhibited 45% α-helix and 5% β-strand content. All EcGrx3 mutants showed reduced α-helical content compared to C66Y, ranging from 15% to 29%. In particular, K19L exhibited the lowest α-helical content (15%), while K19A retained 27%. F29P and F29Y both showed similar, though reduced, α-helical content (27 and 29%, respectively), and the double mutant K19L/F29P demonstrated an even greater reduction (22%). These findings suggest that Phe29 and the Lys19-Glu31 salt bridge are crucial for maintaining EcGrx3′s secondary structure, consistent with the far-UV CD spectroscopy results of SpGrx3 mutants.

**Figure 6:**
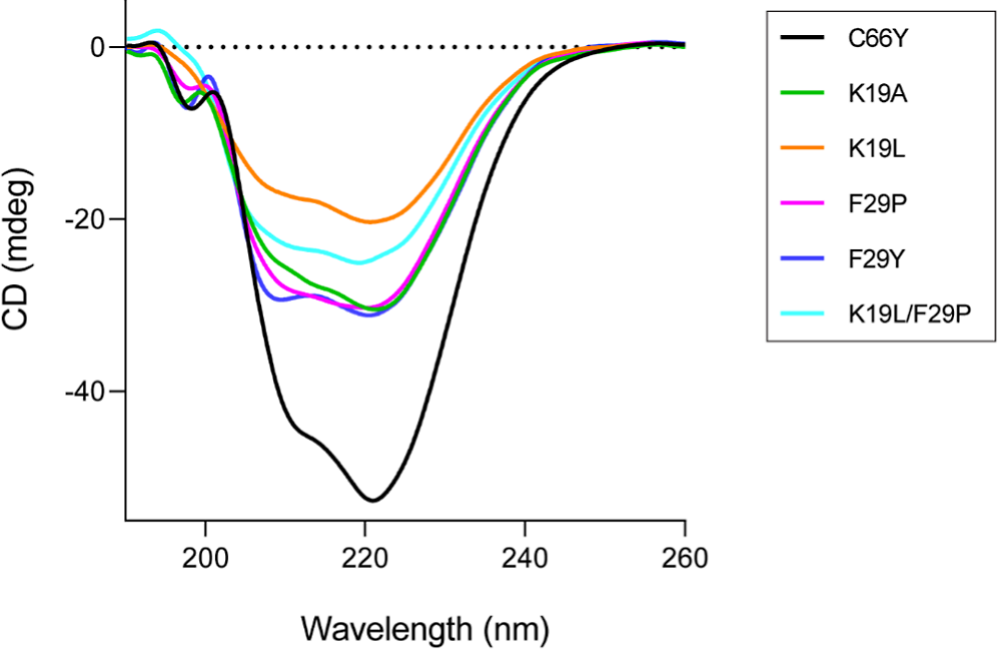
Far-UV CD spectra of EcGrx3 C66Y and its mutants at 25 °C.

### 3.10 MD Simulations of EcGrx3

MD simulations examined the conformational flexibility of EcGrx3 reciprocal mutants (Figure 7). Over 200 ns, C66Y, K19L, and F29P reached relatively stable RMSD values of 1.7 Å, 1.2 Å, and 1.6 Å, respectively. K19L, which disrupts the α1−β2 salt bridge, showed slightly lower fluctuations in loop regions of the aliphatic cluster (β1 loop, α1 loop, α1 helix, and α3 helix) compared to C66Y, indicating that Leu19 and Phe29 hydrophobicity contribute to stability. The highest RMSF values occurred at Gly36 for F29P and C66Y, with F29P at 1.9 Å and C66Y at 1.7 Å, while K19L reached a maximum RMSF at Ala38 (1.8 Å). In the α1 loop, F29P had significantly higher fluctuations than C66Y and K19L, suggesting that replacing Phe29 with Pro disrupts the salt bridge and reduces hydrophobic stabilization.

**Figure 7:**
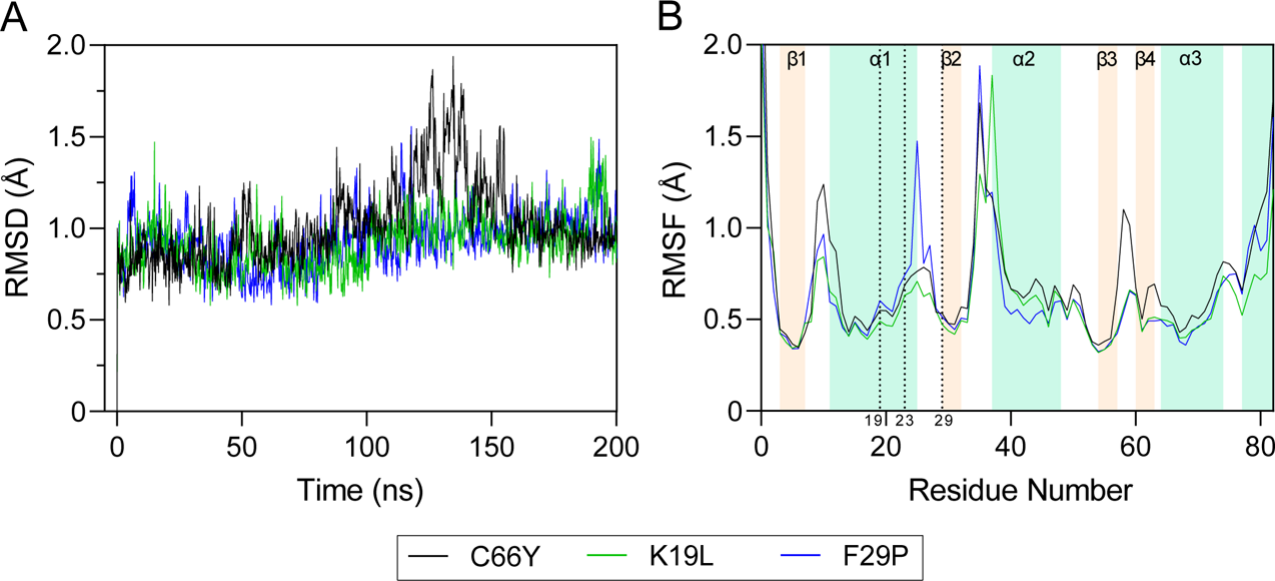
RMSD and RMSF analysis of EcGrx3 C66Y and its mutants. (A) *C*_α_ RMSD over time. (B) *C*_α_ RMSF per residue, with secondary structure elements annotated.

## 4 Discussion

Polar bacteria face various cold-induced stresses in environments ranging from −40 to 0 °C in winter and −10 to 10 °C in summer.^24^ Psychrophilic enzymes adapt by increasing flexibility through reduced intramolecular interactions, enhanced active-site accessibility, longer loops, and higher Gly/Pro and Lys/Arg ratios.^23–26^ These modifications result in lower enthalpy and more negative activation entropy compared to enzymes in warmer conditions.^36,37^ Furthermore, even a single amino acid change in flexible regions—outside the active site—can significantly affect catalysis.^38–40^

Given Grx3′s compact size and ancient Trx fold (over four billion years old),^41,42^ its cold adaptation is especially intriguing. In modern EcTrx, the central β-sheet remains rigid—even in psychrophiles—while the short α3 helix becomes more flexible and the α4 helix becomes more rigid.^16,43,44^ In contrast, structural changes in Grx3 occur mainly on the aromatic cluster side,^17–19^ with increased α2 helix flexibility mirroring that of Trx’s α3 helix.^15,20^

Our data show that Grx3 orthologs adapt to different habitat temperatures by modifying α1−β2 interactions in the aliphatic cluster. Although EcTrx’s aliphatic cluster tolerates nonconservative substitutions,^45,46^ our results show that charged residues in SpGrx3′s aliphatic cluster destabilize the structure. For example, the L19K mutation causes a 3.1 nm red shift in fluorescence, indicating reduced hydrophobic interactions (Figure S8),^47,48^ and an unpaired Lys23 in L19K/A23K/P29F further destabilizes the protein compared to L19K/P29F.

Our results also indicate that the Phe-to-Pro or Tyr-to-Pro change at position 29 is a key adaptation in psychrophilic orthologs, as it disrupts the α1−β2 salt bridge normally present in warmer-temperature orthologs. In SpGrx3, Leu19 and Phe29 stabilize the protein through hydrophobic interactions, whereas Tyr29 stabilizes mainly hydrogen bonding with Asp or Lys at position 23—albeit with lower hydrophobic contribution. Notably, the Lys23-Tyr29 pairing leads to higher *T*_m_ and increased rigidity compared to the Asp23-Tyr29 pair (Table 1, Figure 3), even though urea unfolding experiments indicate that the Lys23-Tyr29 interaction is less stable (Table S2, Figure 2), likely due to Lys’s higher urea contact coefficient (*C*_uw_ = 1.26) relative to Asp (*C*_uw_ = 0.89).^49^ Some psychrophilic orthologs, such as CpGrx3, exhibit the intraloop Lys23-Tyr29 hydrogen bond without the salt bridge.

These findings reveal that Grx3 adapts to cold conditions by replacing the α1−β2 salt bridge with alternative stabilizing interactions. A similar substitution has been observed in glutathione S-transferase (GST), which features an N-terminal Trx-fold domain linked to an all-α-helical C-terminal domain by a short linker, with a critical interaction hotspot in the cleft between the two domains.^50,51^ Thermophilic and mesophilic GST orthologs exhibit highly conserved salt bridge interactions between α1 and the linker loop, and between α3 and α4 (e.g., TtGST, MsGST, EcGST, McGST; Figure S9), whereas psychrophilic GST orthologs (e.g., SpGST, CpGST) rely on hydrophobic interactions in this region.

We reconstructed the predicted pathways for Grx3 cold adaptation based on habitat temperatures and our findings. Although no ancestral Grx3 structures are available, we propose that the ancestral protein contained both the Lys19-Glu31 salt bridge and an intraloop Lys23-Tyr29 hydrogen bond (Figure 8A). Hydrophobic forces peak between 30 and 80 °C and weaken outside this range.^52,53^ Tyr, with its phenolic hydroxyl group, is favored over purely hydrophobic Phe because it forms hydrogen bonds at both low and high temperatures (Figure 8A).^54^ Our model—based on results from SpGrx3 as the psychrophilic enzyme and EcGrx3 as the mesophilic counterpart—illustrates that structural adaptation occurs primarily via Pathway 1, while Pathways 2 and 3 capture additional deviations observed in psychrophilic Grx3 that maintain the hydrogen bond or both the hydrogen bond and salt bridge (Figures 8F–H). While these pathways account for much of the temperature adaptation in Grx3 orthologs, some exceptions remain that cannot be fully explained by our model.

**Figure 8:**
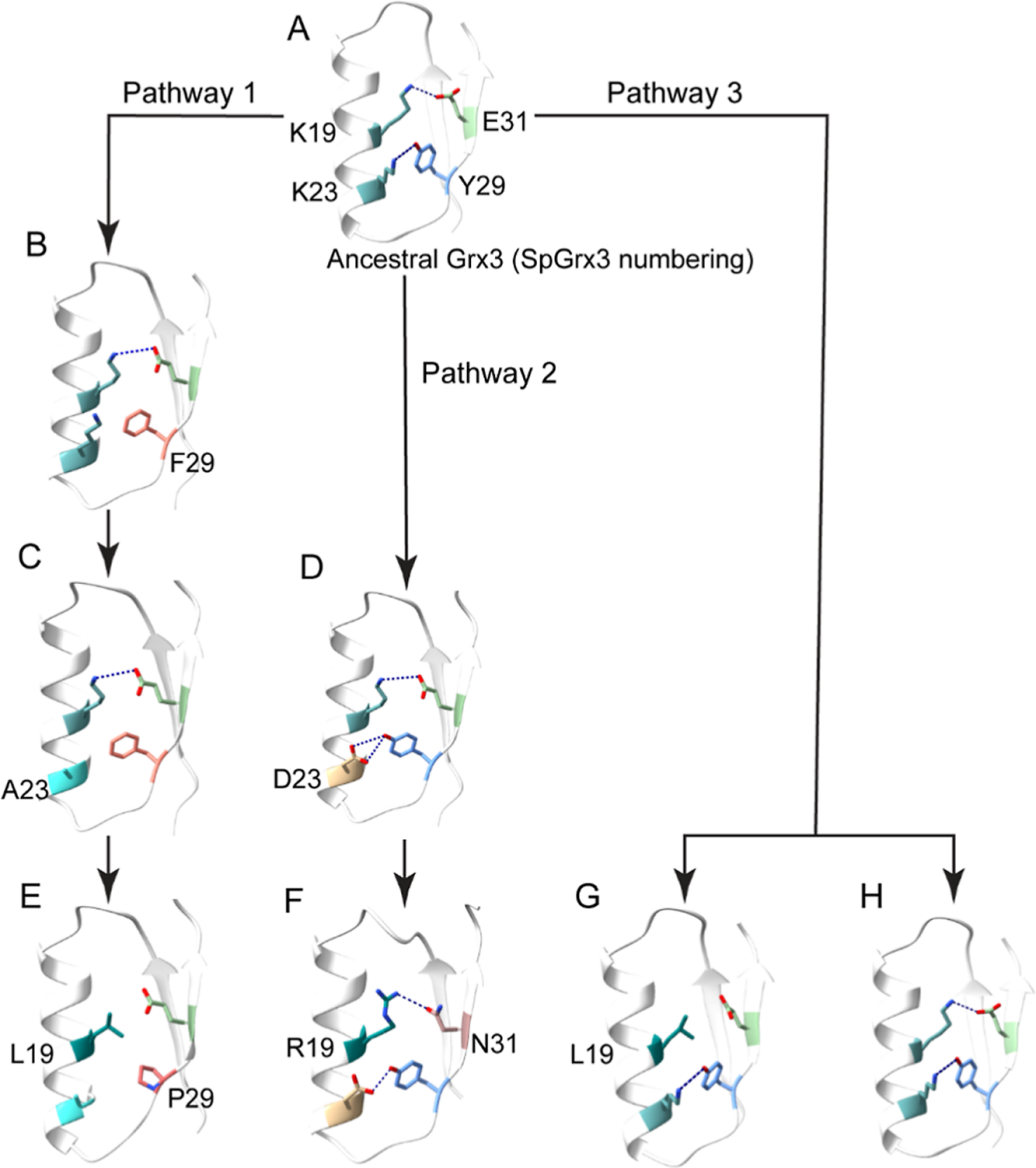
Predicted pathways of Grx3 adaptation to colder temperatures. (A) The predicted ancestral Grx3 possessed both the Lys19-Glu31 salt bridge and the Lys23-Tyr29 hydrogen bond. Pathway 1: As Grx3 evolved to cooler climates, it first underwent a Y29F substitution (B), leaving Lys23 unpaired (as seen in thermophilic Grx3, e.g., CoGrx3). Later, mesophilic orthologs, such as StGrx3, acquired a K23A substitution (C), and psychrophilic orthologs eventually adopted an F29P substitution that disrupted the salt bridge, followed by a K19L change (E) (e.g., SpGrx3). Pathway 2: Some mesophilic Grx3s acquired a K23D substitution (D), forming an Asp23-Tyr29 hydrogen bond while retaining the salt bridge (e.g., DeGrx3). In this pathway, psychrophilic orthologs replaced the salt bridge with an Arg19-Asn31 hydrogen bond (F) (e.g., PhGrx3). Pathway 3: Certain psychrophilic orthologs diverged directly from the ancestral Grx3 without intermediate mesophilic forms. These either feature Leu19 with an intraloop Lys23-Tyr29 hydrogen bond (G) (e.g., CpGrx3) or retain both the salt bridge and Lys23-Tyr29 hydrogen bond (H) (e.g., PsGrx3).

Pathway 1 begins with a Y29F substitution in ancestral Grx3, which leaves Lys23 unpaired, as seen in thermophilic orthologs (e.g., CoGrx3; Figure 8B). In SpGrx3′s configuration mimicking thermophilic orthologs (L19K/A23K/P29F), the unpaired Lys23 increases catalytic efficiency, indicating that high-temperature adaptation favors enzyme activity over stability. Subsequently, mesophilic orthologs such as StGrx3 acquire a K23A substitution (Figure 8C), which improves stability by replacing the unpaired, charged Lys with a small, hydrophobic Ala. EcGrx3 also follows this pathway but features a Ser substitution at this position. Many psychrophilic orthologs, including SpGrx3, then exhibit further structural changes with the K19L/F29P combination, where F29P disrupts the salt bridge to facilitate cold adaptation and K19L enhances stability via hydrophobic interactions (Figure 8E).

Pathway 2 involves an α1-loop K23D substitution in ancestral Grx3, which creates an Asp23-Tyr29 hydrogen bond while maintaining the Lys19-Glu31 salt bridge in mesophilic orthologs (e.g., DeGrx3; Figure 8D). In psychrophilic orthologs (e.g., PhGrx3 from Pseudoalteromonas haloplanktis), the salt bridge is replaced by an Arg19-Gln31 hydrogen bond, though the Asp23-Tyr29 hydrogen bond remains (Figure 8F). While most Grx3 proteins retain a conserved GSH binding site regardless of temperature, PhGrx3 replaces Arg51 with Ala in the α2 helix, increasing the flexibility of its GSH binding site.^20^

Pathway 3 includes psychrophilic orthologs that appear to have diverged directly from the ancestral form. Although the transition to Pro29 is critical for cold adaptation, the modest 1 °C *T*_m_ difference between the Lys19-Glu31 salt bridge and Leu19 hydrophobic interactions—both with the Lys23-Tyr29 hydrogen bond—suggests that these stabilization mechanisms could provide comparable protection. However, the occurrence of the Leu19 combination is rare (e.g., CpGrx3; Figure 8G), suggesting that this alternative mechanism is not commonly favored. In contrast, PsGrx3 from the Antarctic bacterium *Psychrobacter* sp. retains the ancestral configuration (Figure 8H) and, like PhGrx3 (Figure 8F), substitutes Arg51 with Tyr in the α2 helix and His63 with Phe in the β4 strand.^22^

Overall, these pathways suggest that Grx3 adapts to cold through different structural modifications, with Pathway 1 being the most common and the Pathway 3 representing the most parsimonious evolutionary route.

Pro, with its secondary amine group, is often found in protein turns and loops—especially in α–β loops^55,56^ —but its effect on stability depends on its location. Pro at the N-terminus of α-helices enhances stability, while Pro in the middle increases flexibility.^57,58^ Moreover, cis-Pro induces a more compact conformation than trans-Pro.^59^ In the evolution from thermophilic to psychrophilic Grx3, substituting Phe29 with Pro at the end of the α1 loop does not enhance stability but accelerates cold adaptation. Although increased flexibility is a common cold adaptation strategy,^26,27^ psychrophilic orthologs in Pathways 2 and 3 retain both the salt bridge and the Tyr29 hydrogen bond—features reminiscent of ancestral Grx3—suggesting that adaptations in these pathways may occur in regions other than the α1−β2 interface, such as the GSH binding site, as seen in PhGrx3 (Figure 8F) and PsGrx3 (Figure 8H).^20^

The phylogenetic tree was constructed in MEGA X using 106 Grx3 sequences (16 eukaryotic, 9 archaeal, and 81 bacterial). No ancestor sequence was reconstructed, and the tree shows that temperature adaptation evolved independently within each clade. Each clade includes orthologs from a range of temperatures—from thermophilic to psychrophilic (Figure S10). Based on protein structure, the orthologs in pathway 1 (CoGrx3, StGrx3, and SpGrx3) are located in distinct clades. In contrast, PsGrx3, which exhibits the predicted ancestral configuration (Figure 8A,H), clusters with an archaeal clade (Figure S10), suggesting it has retained ancestral features that support the predicted α1−β2 interactions. Other archaeal clades indicate structural diversity within Archaea, while eukaryotic orthologs form a separate lineage from prokaryotic ones.

Although the Trx fold is highly conserved, individual family members have evolved distinct cold adaptations reflecting differences in structure and function, including variations in the number of α/β-secondary structures and enzyme function.^16,43,54,60,61^ Our study provides new insights into Grx3′s structural adaptations in its aliphatic cluster—a region less studied than the aromatic cluster. Grx3 proteins use diverse α1−β2 interactions for temperature adaptation, offering new perspectives on how other Trx-fold proteins adjust to temperature changes.

## Supplementary Material



## Notes

List of primers for site-directed mutagenesis, conformational stability parameters, inverse Stern–Volmer quenching constant of SpGrx3 WT and mutants, secondary structure analysis of SpGrx3 and EcGrx3, structural model of Grx3 orthologs, effect of temperature on SpGrx3 activity assay, SDS-PAGE analysis of SpGrx3 and EcGrx3, size-exclusion chromatography analysis of SpGrx3 WT and mutants, thermal denaturation curves of SpGrx3 and EcGrx3, intrinsic fluorescence of SpGrx3 WT and L19K mutant, structural model of the cleft between N- and C-terminal domain of GSTs, and phylogenetic tree based on the amino acid sequence of eukaryotic, archaeal, and bacterial Grx3 (PDF)

## Abbreviations

GSH: reduced glutathione
EDTA: ethylenediaminetetraacetic acid
HED: hydroxyethyl disulfide
RMSD: root-mean-square deviation
RMSF: root-mean-square fluctuation
