# Supplementary material for: Structural Adaptations of Bacterial Grx3 to Temperature: Pro29 Is Essential for Cold Adaptation in Sphingomonas sp. Grx3
Source: ACS Omega. 2025 May 26;10(22):23848–57. doi: 10.1021/acsomega.5c03414 (PMC12163845; doi:10.1021/acsomega.5c03414)
Supplement: Supplementary file 1 [file ao5c03414_si_001.pdf]

Supporting Information for:

Structural Adaptations of Bacterial Grx3 to Temperature: Pro29 Is Essential for Cold Adaptation in *Sphingomonas* sp. Grx3

Luyen Vu [1], ChangWoo Lee [1,] *

[1]Department of Biomedical Science and Center for Bio-Nanomaterials, Daegu University, Gyeongsan 38453, South Korea

*To whom correspondence should be addressed: Email: leec@daegu.ac.kr Tel: +82-53-850-6464.

Table S1. List of Primers for Site-Directed Mutagenesis

		Forward primer (5′→3′)	Reverse primer (5′→3′)
SpGrx3	L19K	cgcgcgcgAAgaagctg	cgagcagcttcTTcgcgc
	P29F	cacgatgggcggcTATaagcg	tttcggtgcgcttATAgccgc
	P29Y	aagggtgtcacgTATgaagag	gatgtcatactcttcATAcgtgac
	A23D	ctgaagctgctcgACtcgaag	gacacccttcgaGTcgagc
	A23K	gcgctgaagctgctcAAgtcg	cgtgacacccttcgacTTgagcag
EcGrx3	C66Y	gcacattggcggctAtgatg	gcgcatacaaatcatcaTagccg
	K19A	ccatattgtcatcgtgctGCTgc	gagctcaggagtgcAGCagc
	K19L	ccatattgtcatcgtgctTTagcac	gagctcaggagtgctAAagcac
	F29P	ctaaaggggtgagtCCccaag	gataggtaactcttggGGactcac
	F29Y	ctaaaggggtgagttAccaag	cgataggtaactcttggTaactc

Mutated codons have upper-case letters.

Table S2. Conformational Stability Parameters of SpGrx3 WT and Mutants

	$[D]_{½}$ (M)	m_{NU} [b] (kcal mol^{-1} M^{-1})	$\Delta G°'_{H2O}$ [c] (kcal mol^{-1})
WT	3.1 ± 0.3	0.8 ± 0.1	2.3 ± 0.1
L19K	2.4 ± 0.1	0.5 ± 0.1	1.1 ± 0.1
P29F	5.0 ± 0.2	0.6 ± 0.0	3.1 ± 0.2
P29Y	4.6 ± 0.2	0.5 ± 0.0	2.3 ± 0.1
L19K/P29F	5.8 ± 0.3	0.4 ± 0.0	2.5 ± 0.3
L19K/P29Y	5.1 ± 0.1	0.4 ± 0.0	1.8 ± 0.2
A23D/P29Y	5.5 ± 0.3	0.6 ± 0.0	3.4 ± 0.0
A23K/P29Y	5.3 ± 0.1	0.4 ± 0.1	2.1 ± 0.3
L19K/A23D/P29Y	6.1 ± 0.1	0.5 ± 0.0	2.8 ± 0.2
L19K/A23K/P29Y	5.2 ± 0.1	0.5 ± 0.0	2.4 ± 0.2
L19K/A23K/P29F	5.7 ± 0.2	0.4 ± 0.1	2.5 ± 0.5

[a] Concentration of urea at which the unfolding transition midpoint is observed.
[b] Proportionality constant between free energy and urea concentration.
[c] Free energy of unfolding extrapolated to zero denaturants.
Data presented are the means ± SD of three experiments.

Table S3. Inverse Stern-Volmer Quenching Constant, K_{sv}^{-1}, of SpGrx3 WT and Mutants.

	K_{sv}^{-1} (mM)
WT	39.3 ± 2.0
L19K	38.6 ± 3.1
P29F	43.0 ± 2.6
P29Y	51.7 ± 2.0
L19K/P29F	70.4 ± 2.3
L19K/P29Y	55.7 ± 3.9
A23D/P29Y	52.3 ± 3.9
A23K/P29Y	54.5 ± 3.5
L19K/A23D/P29Y	50.1 ± 2.1
L19K/A23K/P29Y	56.9 ± 2.6
L19K/A23K/P29F	49.7 ± 1.3

K_{sv}^{-1} is the acrylamide concentration at which 50% of the fluorescence intensity is quenched. Data presented are the means ± SD of three experiments.

Table S4. Content of α-Helix and β-Strand in SpGrx3 WT, EcGrx3 C66Y, and Their Mutants.

		α-helix (%)	β-strand (%)
SpGrx3	WT	35	11
	L19K	18	17
	P29F	11	19
	P29Y	11	19
	L19K/P29F	6	25
	L19K/P29Y	8	22
	A23D/P29Y	8	23
	A23K/P29Y	7	23
	L19K/A23D/P29Y	5	25
	L19K/A23K/P29Y	25	14
	L19K/A23K/P29F	10	21
EcGrx3	C66Y	45	5
	K19A	27	12
	K19L	15	16
	F29P	27	12
	F29Y	29	11
	K19L/F29P	22	14

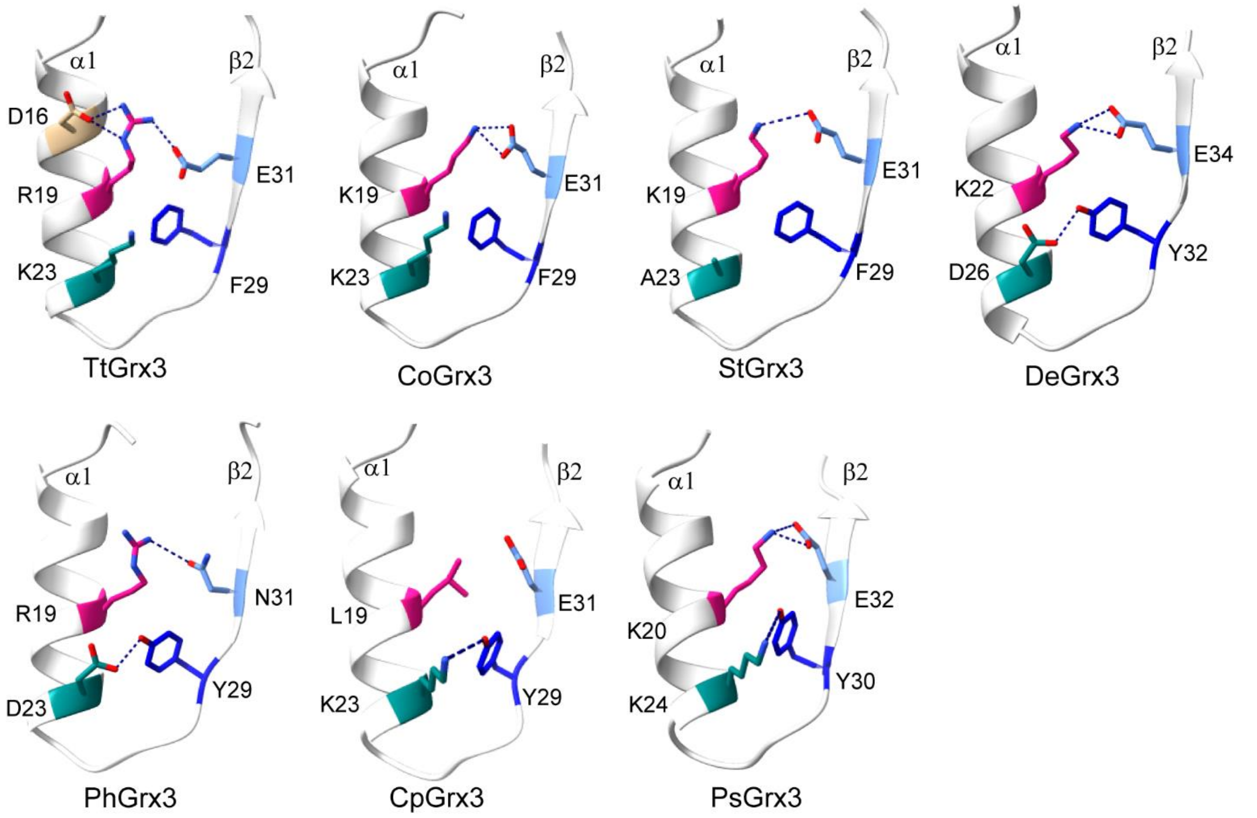

Figure S1. Enlarged view of α1-β2 interactions in Grx3 orthologs. Thermophilic Grx3s: TtGrx3 (*Thermochromatium tepidum* ATCC 43061, NCBI: QGU33019.1) and CoGrx3 (*Chromatium okenii*, NCBI: WP_105074337.1); Mesophilic Grx3s: StGrx3 (*Sphingosinithalassobacter tenebrarum* zrk23, NCBI: WP_165327942.1) and DeGrx3 (*Desulfuromonadales* bacterium, NCBI: NIQ93024.1); Psychrophilic Grx3s: PhGrx3 (*Pseudoalteromonas haloplanktis* TAC125, NCBI: YP_338909.1), CpGrx3 (*Colwellia piezophila*, NCBI: WP_019027132.1), and PsGrx3 (*Psychrobacter* sp. ANT206, NCBI: QUQ60702.1).

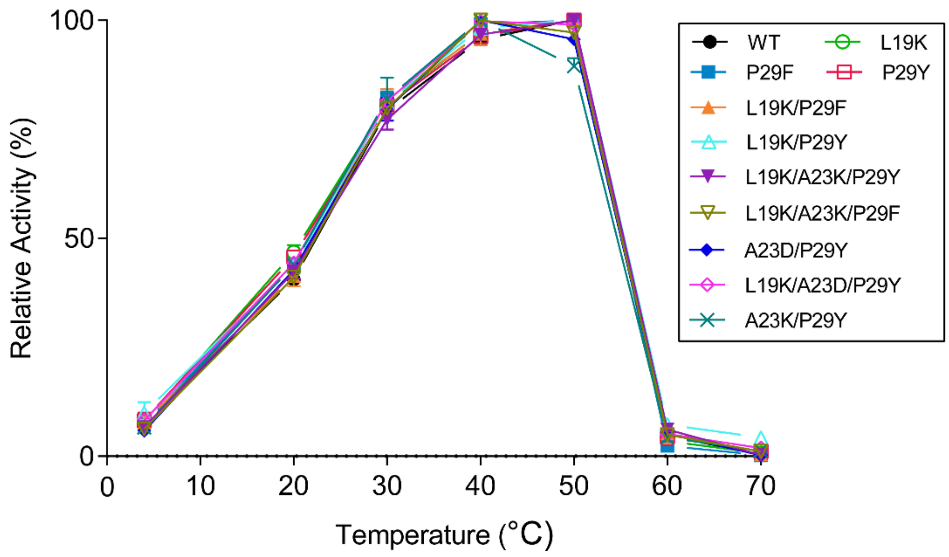

Figure S2. Effect of temperature on SpGrx3 activity. Data presented are the means ± SD of three experiments.

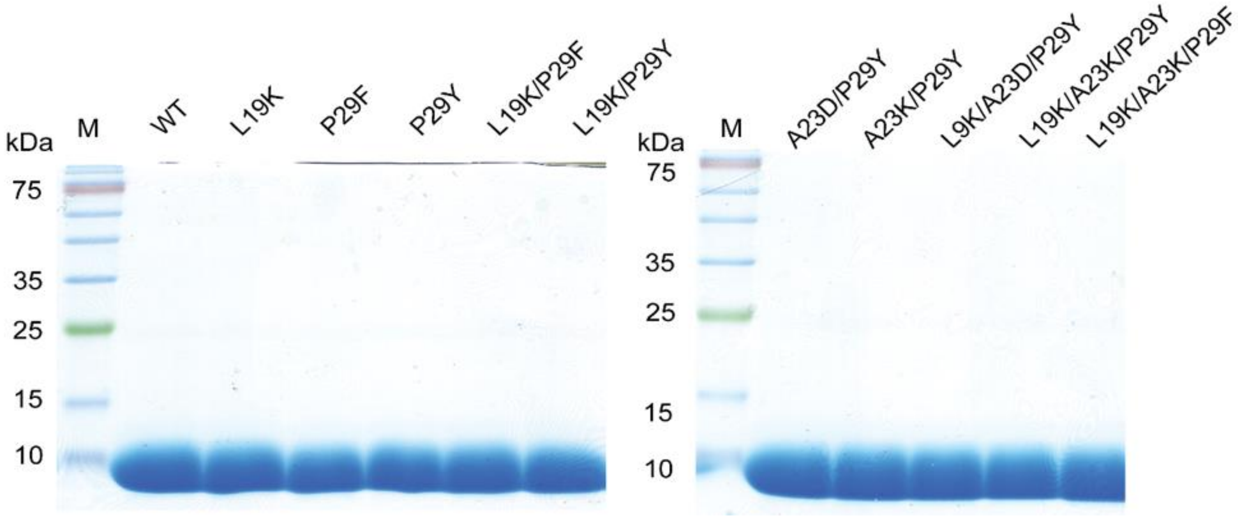

Figure S3. SDS-PAGE analysis of SpGrx3 WT and mutants.

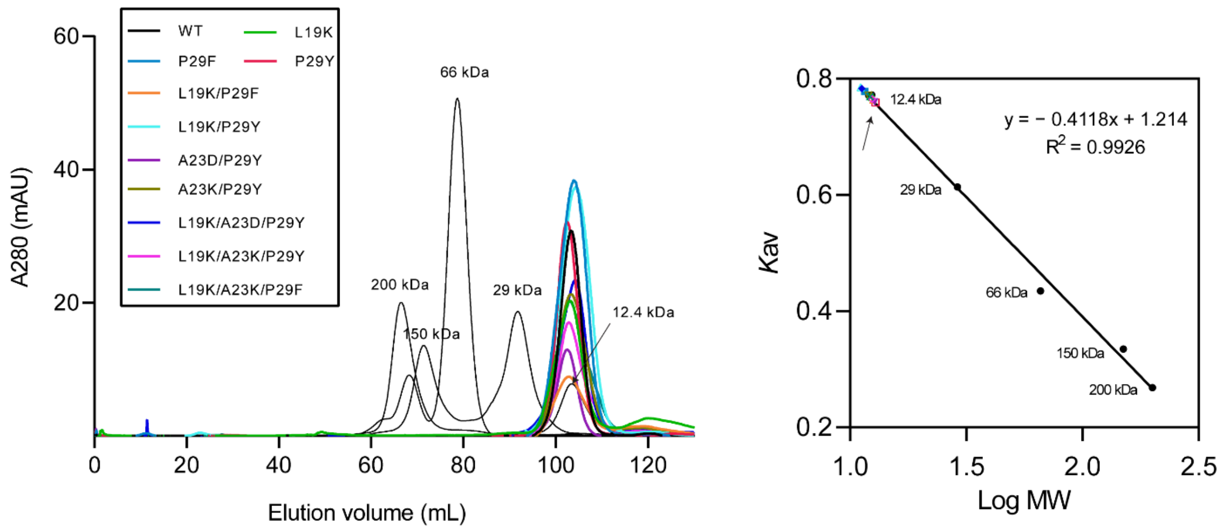

Figure S4. Size-exclusion chromatography analysis of SpGrx3 WT and mutants. Arrows indicate that the estimated MWs range from 11.1 to 12.8 kDa.

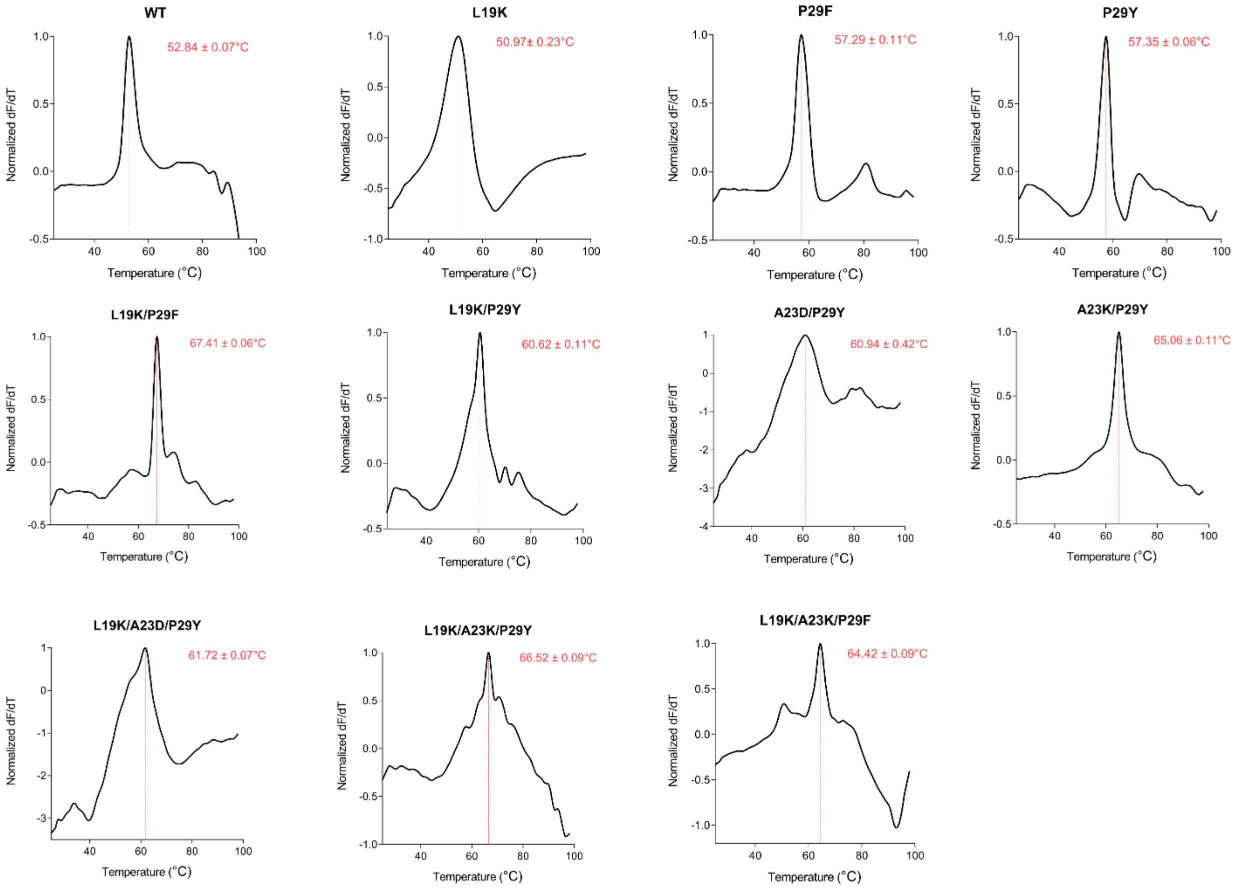

Figure S5. Melting temperature of SpGrx3 WT and mutants. Data represents the mean ± SD of three experiments.

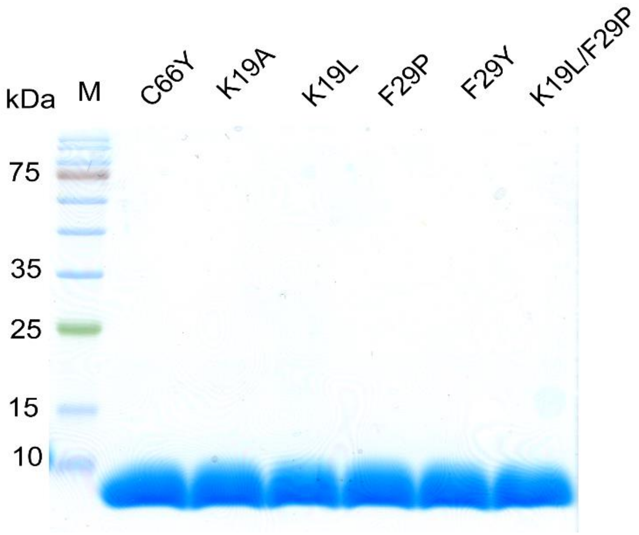

Figure S6. SDS-PAGE analysis of EcGrx3 C66Y and its mutants. M, marker.

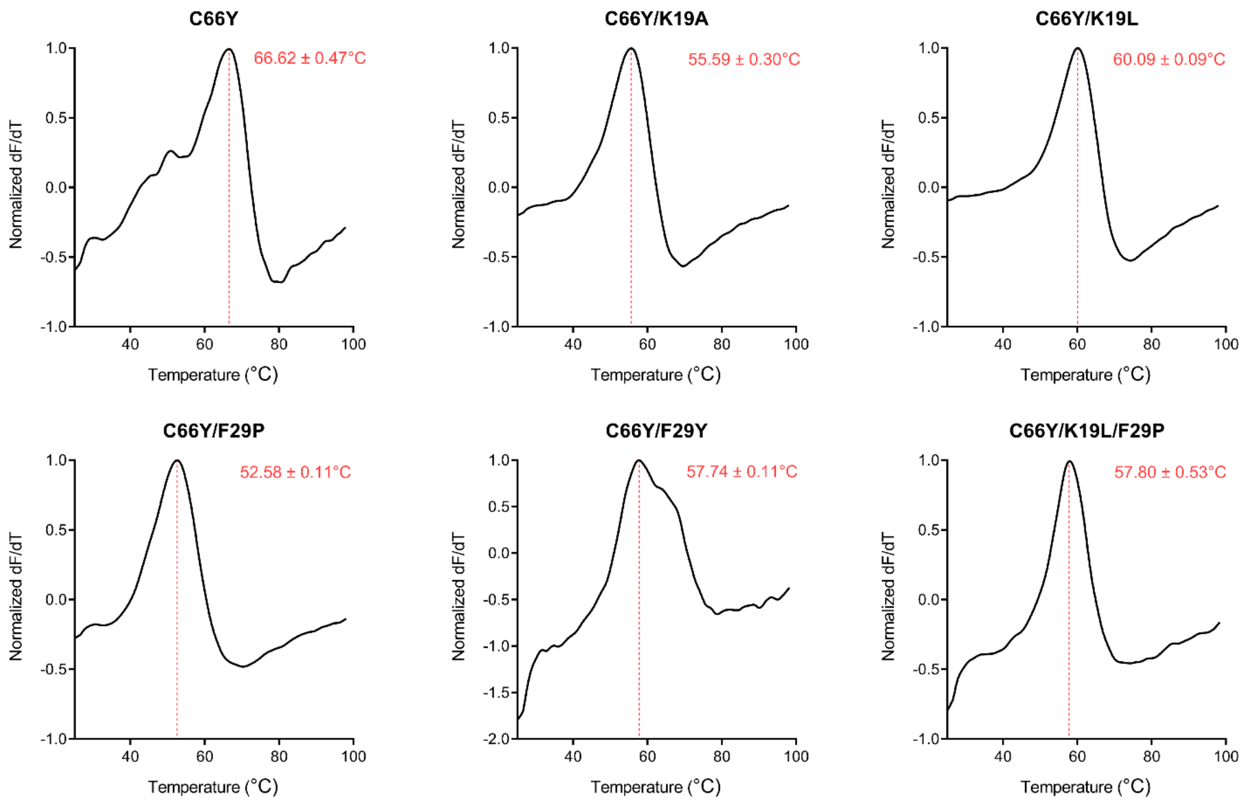

Figure S7. Melting temperatures of EcGrx3 C66Y and its mutants. Data represents the mean ± SD of three experiments.

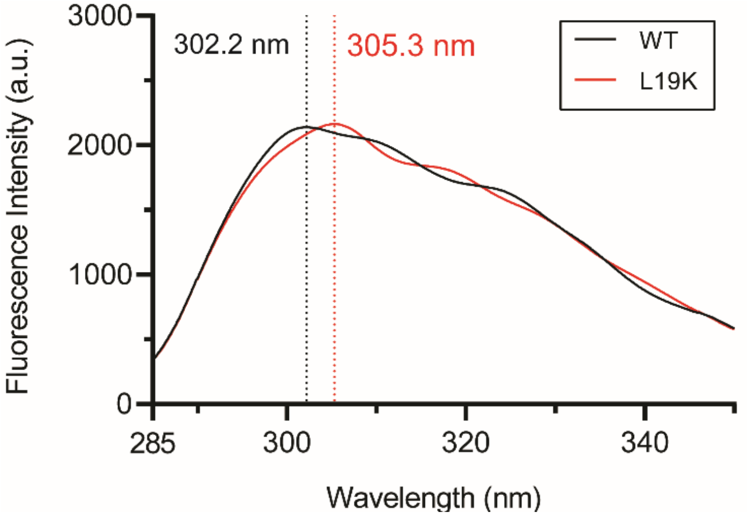

Figure S8. Intrinsic fluorescence of SpGrx3 WT and L19K**.** Data presented are the mean ± SD of three experiments.

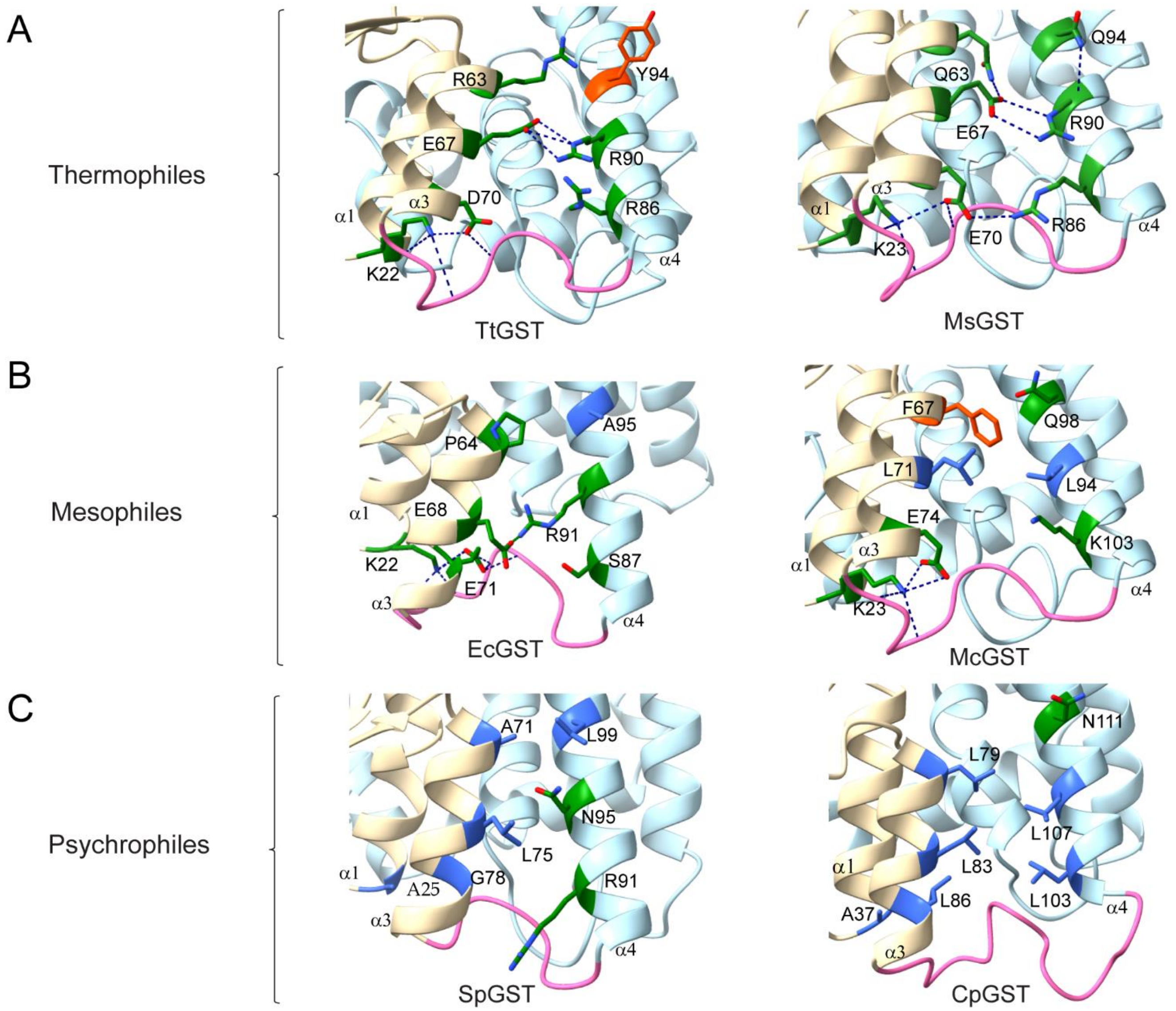

Figure S9. Enlarged view of the cleft between N- and C-terminal domains in GSTs**.** The N-terminal domain and C-Terminal domains are shown in distinct colors, while the linker sequence is depicted in pink. (A) Thermophilic GSTs: TtGST (*Thermochromatium tepidum* ATCC 43061, NCBI ID: WP_153975431.1), MsGST (*Methylococcus* sp. Mc7, NCBI ID: WP_218807172.1). (B) Mesophilic GSTs: EcGrx3 (*Escherichia coli* K-12, NCBI ID: QPD61329.1), McGST (*Moraxella catarrhalis* 25239, NCBI ID: AIT43523.1). (C) Psychrophilic GSTs: SpGST (*Sphingomonas* sp. PAMC 26621, NCBI ID: WP_026046435.1), CpGST (*Colwellia piezophila* ATCC BAA-637, NCBI ID: WP_019029435.1).

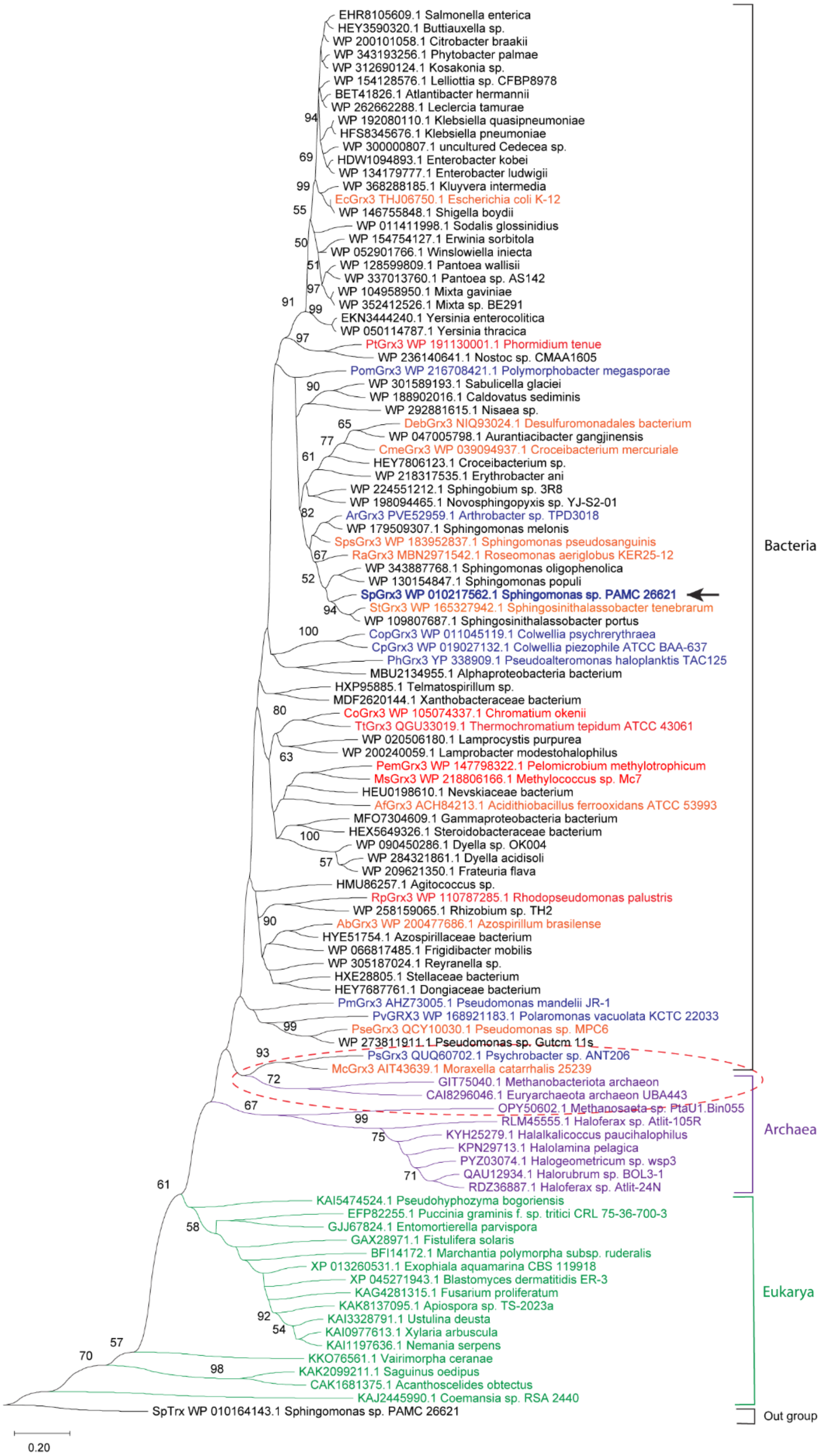
EHR8105609.1 Salmonella enterica
HEY3590320.1 Buttiauxella sp.
WP 200101058.1 Citrobacter braakii
WP 343193256.1 Phytobacter palmae
WP 312690124.1 Kosakonia sp.
WP 154128576.1 Lelliottia sp. CFBP8978
BET41826.1 Atlantibacter hermannii
WP 262662288.1 Leclercia tamurae
WP 192080110.1 Klebsiella quasipneumoniae
HFS8345676.1 Klebsiella pneumoniae
WP 300000807.1 uncultured Cedecea sp.
HDW1094893.1 Enterobacter kobei
WP 134179777.1 Enterobacter ludwigii
WP 368288185.1 Kluyvera intermedia
EcGrx3 THJ06750.1 Escherichia coli K-12
WP 146755848.1 Shigella boydii
WP 011411998.1 Sodalis glossinidius
WP 154754127.1 Erwinia sorbitola
WP 052901766.1 Winslowiella iniecta
WP 128599809.1 Pantoea wallisii
WP 337013760.1 Pantoea sp. AS142
WP 104958950.1 Mixta gaviniae
WP 352412526.1 Mixta sp. BE291
EKN3444240.1 Yersinia enterocolitica
WP 050114787.1 Yersinia thracica
PtGrx3 WP 191130001.1 Phormidium tenue
WP 236140641.1 Nostoc sp. CMAA1605
PomGrx3 WP 216708421.1 Polymorphobacter megasporae
WP 301589193.1 Sabulicella glaciei
WP 188902016.1 Caldovatus sediminis
WP 292881615.1 Nisaea sp.
DebGrx3 NIQ93024.1 Desulfuromonadales bacterium
WP 047005798.1 Aurantiacibacter gangjinensis
CmeGrx3 WP 039094937.1 Croceibacterium mercuriale
HEY7806123.1 Croceibacterium sp.
WP 218317535.1 Erythrobacter ani
WP 224551212.1 Sphingobium sp. 3R8
WP 198094465.1 Novosphingopyxis sp. YJ-S2-01
ArGrx3 PVE52959.1 Arthrobacter sp. TPD3018
WP 179509307.1 Sphingomonas melonis
SpsGrx3 WP 183952837.1 Sphingomonas pseudosanguinis
RaGrx3 MBN2971542.1 Roseomonas aeriglobus KER25-12
WP 343887768.1 Sphingomonas oligophenolica
WP 130154847.1 Sphingomonas populi
SpGrx3 WP 010217562.1 Sphingomonas sp. PAMC 26621
StGrx3 WP 165327942.1 Sphingosinithalassobacter tenebrarum
WP 109807687.1 Sphingosinithalassobacter portus
CopGrx3 WP 011045119.1 Colwellia psychrerythraea
CpGrx3 WP 019027132.1 Colwellia piezophile ATCC BAA-637
PhGrx3 YP 338909.1 Pseudoalteromonas haloplanktis TAC125
MBU2134955.1 Alphaproteobacteria bacterium
HXP95885.1 Telmatospirillum sp.
MDF2620144.1 Xanthobacteraceae bacterium
CoGrx3 WP 105074337.1 Chromatium okenii
TtGrx3 QGU33019.1 Thermochromatium tepidum ATCC 43061
WP 020506180.1 Lamprocystis purpurea
WP 200240059.1 Lamprobacter modestohalophilus
PemGrx3 WP 147798322.1 Pelomicrobium methylotrophicum
MsGrx3 WP 218806166.1 Methylococcus sp. Mc7
HEU0198610.1 Nevskiaceae bacterium
AfGrx3 ACH84213.1 Acidithiobacillus ferrooxidans ATCC 53993
MFO7304609.1 Gammaproteobacteria bacterium
HEX5649326.1 Steroidobacteraceae bacterium
WP 090450286.1 Dyella sp. OK004
WP 284321861.1 Dyella acidisoli
WP 209621350.1 Frateuria flava
HMU86257.1 Agitococcus sp.
RpGrx3 WP 110787285.1 Rhodopseudomonas palustris
WP 258159065.1 Rhizobium sp. TH2
AbGrx3 WP 200477686.1 Azospirillum brasilense
HYE51754.1 Azospirillaceae bacterium
WP 066817485.1 Frigidibacter mobilis
WP 305187024.1 Reyranella sp.
HXE28805.1 Stellaceae bacterium
HEY7687761.1 Dongiaceae bacterium
PmGrx3 AHZ73005.1 Pseudomonas mandelii JR-1
PvGRX3 WP 168921183.1 Polaromonas vacuolata KCTC 22033
PseGrx3 QCY10030.1 Pseudomonas sp. MPC6
WP 273811911.1 Pseudomonas sp. Gutcm 11s
PsGrx3 QUQ60702.1 Psychrobacter sp. ANT206
McGrx3 AIT43639.1 Moraxella catarrhalis 25239
GIT75040.1 Methanobacteriota archaeon
CAI8296046.1 Euryarchaeota archaeon UBA443
OPY50602.1 Methanosaeta sp. PtaU1.Bin055
RLM45555.1 Haloferax sp. Atlit-105R
KYH25279.1 Halalkalicoccus paucihalophilus
KPN29713.1 Halolamina pelagica
PYZ03074.1 Halogeometricum sp. wsp3
QAU12934.1 Halorubrum sp. BOL3-1
RDZ36887.1 Haloferax sp. Atlit-24N
KAI5474524.1 Pseudohyphozyma bogoriensis
EFP82255.1 Puccinia graminis f. sp. tritici CRL 75-36-700-3
GJJ67824.1 Entomortierella parvispora
GAX28971.1 Fistulifera solaris
BFI14172.1 Marchantia polymorpha subsp. ruderalis
XP 013260531.1 Exophiala aquamarina CBS 119918
XP 045271943.1 Blastomyces dermatitidis ER-3
KAG4281315.1 Fusarium proliferatum
KAK8137095.1 Apiospora sp. TS-2023a
KAI3328791.1 Ustulina deusta
KAI0977613.1 Xylaria arbuscula
KAI1197636.1 Nemania serpens
KKO76561.1 Vairimorpha ceranae
KAK2099211.1 Saguinus oedipus
CAK1681375.1 Acanthoscelides obtectus
KAJ2445990.1 Coemansia sp. RSA 2440
SpTrx WP 010164143.1 Sphingomonas sp. PAMC 26621
Bacteria
Archaea
Eukarya
Out group
0.20

Figure S10. Phylogenetic tree of Grx3 based on 16 eukaryotic, 9 archaeal, and 81 bacterial sequences. Sequences were aligned using MUSCLE and the tree constructed in MEGA X with the WAG+G model, using SpTrx as the outgroup. Bootstrap values (1000 replicates via neighbor-joining) are shown only if ≥50%. Branch colors: eukaryotic (green), archaeal (purple), bacterial (black); within bacteria, thermophilic (red), mesophilic (orange), psychrophilic (blue); species with undetermined temperature preference are shown in black.
